# Genomic Editing: The Evolution in Regulatory Management Accompanying Scientific Progress

**DOI:** 10.3389/fbioe.2022.835378

**Published:** 2022-02-21

**Authors:** María Florencia Goberna, Agustina Inés Whelan, Perla Godoy, Dalia Marcela Lewi

**Affiliations:** National Directorate of Bioeconomy, Secretariat of Food, Bioeconomy and Regional Development, MAGyP, Buenos Aires, Argentina

**Keywords:** genome editing, innovation, regulation, new breeding techniques, modern biotechnology

## Abstract

Argentina currently has a regulation for genome-editing products whose criteria were updated as consultations were received to determine the regulatory status of these products. The aim of this regulation is to consider all organisms (animals, micro-organisms and plants) under the same NBT resolution independently and without being linked to commercial Genetically Modified Organism (GMO) regulations. This gives certainty to local researchers and developers (teams of local developers and researchers), which can be seen in the number of developments and consultations carried out. It should be noted that early results showed that the speed of innovation of these technologies was increasing in a short time, giving more opportunity to local developers who showed interest in generating products in different species, crops and phenotypes.

## Introduction

To begin with, modern biotechnology can be defined as the technological application of genetic engineering tools for the improvement of crops, micro-organisms, and animals of agricultural interest, with the aim of generating benefits for farmers, consumers, industry, human and animal health, and the environment. That is why some of the purposes of this technology are to improve and increase agricultural production, reduce production costs, make more efficient use of resources, promote resilience to climate change while preserving the productive environment, and increase food safety, security and quality (Petracca et al., 2016; Duensing et al., 2018; Gavin et al., 2018; Eriksson et al., 2019).

Argentina, in particular, has increased its production of genetically modified (GM) crops, currently being the world’s third largest producer of biotech crops, after the United States and Brazil with a degree of adoption of transgenic varieties that, in the case of soybean and cotton, represents 99% of total trade with these crops and 98% in the case of maize, demonstrating the high degree of acceptance and adoption of these technologies by Argentine farmers (ISAAA, 2018).^1^

Argentina is one of the first countries to have developed and applied modern biotechnology techniques since the late 1980s (Burachik and Traynor, 2002) being this the basis for the development of a sound regulatory framework, which was set in motion with the creation of the National Advisory Commission on Agricultural Biotechnology (CONABIA) in 1991 which, as an evaluation and consultation body, has a multidisciplinary approach and is composed of experts representing different sectors such as environment, health and agriculture, which is why it is one of the first countries to have developed and applied modern biotechnology techniques (Burachik and Traynor, 2002).

With 30 years of experience in the regulation of products obtained through genetic engineering, Argentina has consolidated its experience and capacity to determine criteria for the biosafety analysis of these products, which are used in the production of pharmaceuticals and in the human and animal food industry.

It is through CONABIA that Argentina provides advice and training, and collaborates with other countries on biotechnology regulatory approaches and frameworks. It is worth noting that due to its trajectory, in 2014 CONABIA was recognised by FAO (Food and Agriculture Organization of the United Nations) as a “Reference Centre” for the Biosafety of Genetically Modified Organisms (GMOs) and its designation was renewed in 2019.

Genomic editing is part of the group of so-called “New Breeding Techniques” (NBT). Like transgenesis, genome editing is a genetic engineering tool whose application allows for more sustainable food production, more nutritious products and better protection of crops against pests, diseases and climatic adversities. The difference between NBTs and GMOs is that these innovative tools allow targeted and precise DNA modifications (Barrangou and Doudna, 2016; Knott and Doudna, 2018; Chen et al., 2019).

Argentina has carried out an update and improvement of its entire regulatory framework for both GMOs and NBTs in 2020. The characteristics of New Breeding Techniques (NBTs) regulatory measures require a prior scientific analysis, on a case-by-case basis, of organisms already obtained or to be obtained, in order to determine whether they fall within the scope of the regulations applicable to Genetically Modified Organisms (GMOs) or not. In other words, the regulatory framework for NBTs establishes the procedures to determine whether or not any organism obtained through new breeding techniques using modern biotechnology is covered by GMO regulations.

### Current Official Regulation

In 2013, Argentina carried out a preliminary analysis on the state of the art of NBTs in the world. Two years later, Argentina officially published the first NBT regulation only for plants (Whelan and Lema, 2015). A few years later, in 2019, the NBT regulation for animals and microorganisms was published and the regulation for plants was updated (Whelan and Lema, 2019).

As mentioned above, during 2020 the NBT regulations was updated and simplified and was officially published in the following year under Resolution N° 21/2021.^2^ This resolution is based on N° 763 of 17 August 2011 (Ministry of Agriculture, Livestock, and Fisheries) which uses the Cartagena Protocol.^3^ definition of GMO, understood as any living organism that possesses a new combination of genetic material obtained through the application of modern biotechnology. In addition, this new resolution includes the definition of “novel combination of genetic material” which refers to any change produced in the genome of the organism by the incorporation, in a stable and cohesive manner, of one or more genes or nucleic acid sequences that are part of a defined genetic construct.

This new NBT Resolution N° 21/2021 takes into account a procedure to determine whether a product obtained by NBT could be covered by the GMO regulation or not. This analysis begins when the interested party completes the Prior Consultation Instance (PCI) form according to the organisms of interest (plant, animal, or micro-organism). It should be clarified that this form can be submitted when the product is finished or when it is in the design stage). In case a PCI has been submitted for a product in the design stage, the developer must submit a second form when the product is finished, in order to verify whether the modifications made are the same as those described in the first PCI.

In this way, the National Directorate of Bioeconomy scientific-technical evaluation team and CONABIA analyze whether the product does not have a new combination of genetic material based on the information submitted. If there is indeed no new combination of genetic material, the product is non-GM and is considered as a conventional product. On the other hand, if the product has a new combination of genetic material, it is considered transgenic and must comply with GMO regulations according to the organism, animal, micro-organism and plant.

The analysis is carried out on a case-by-case basis, it is not limited to a specific list of techniques and allows for consultation when the product is at the design stage. Finally, the Commission must provide a response to the interested party within 80 working days. This updated regulation has specific annexes for animals, microorganisms and plants, as a guide to the information that the developer has to take into account when completing the PCI.

### PCIs Assessment

From the beginning of the application of the above-mentioned criteria, included in the first version of the NBT regulation, it was observed that these measures promoted the submission for consultation of developers (Whelan et al., 2020).

The analysis of the entire experience generated by the application of these regulations reveals the following conclusions about the PCI cases analyzed: 1) the developers can predict costs and period of time in the product development, even at the design stage; 2) the developers can put their products into the market sooner; 3) there are a greater phenotype varieties in different crops, animals and microorganisms; and 4) the speed of innovation of products obtained by NBTs is greater in relation to GMOs the innovation speed.

**FIGURE 1:**
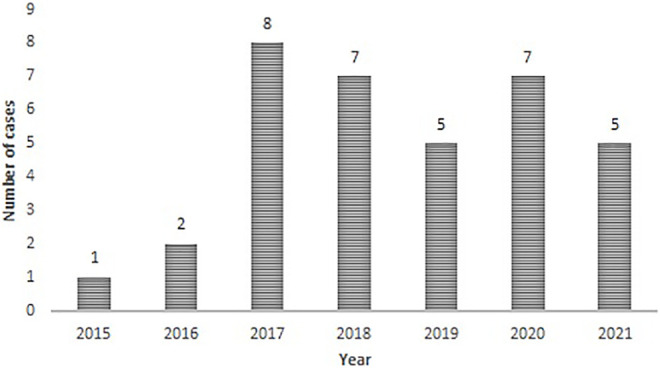
Number of PCI cases analyzed between 2015 and 2021.

**FIGURE 2:**
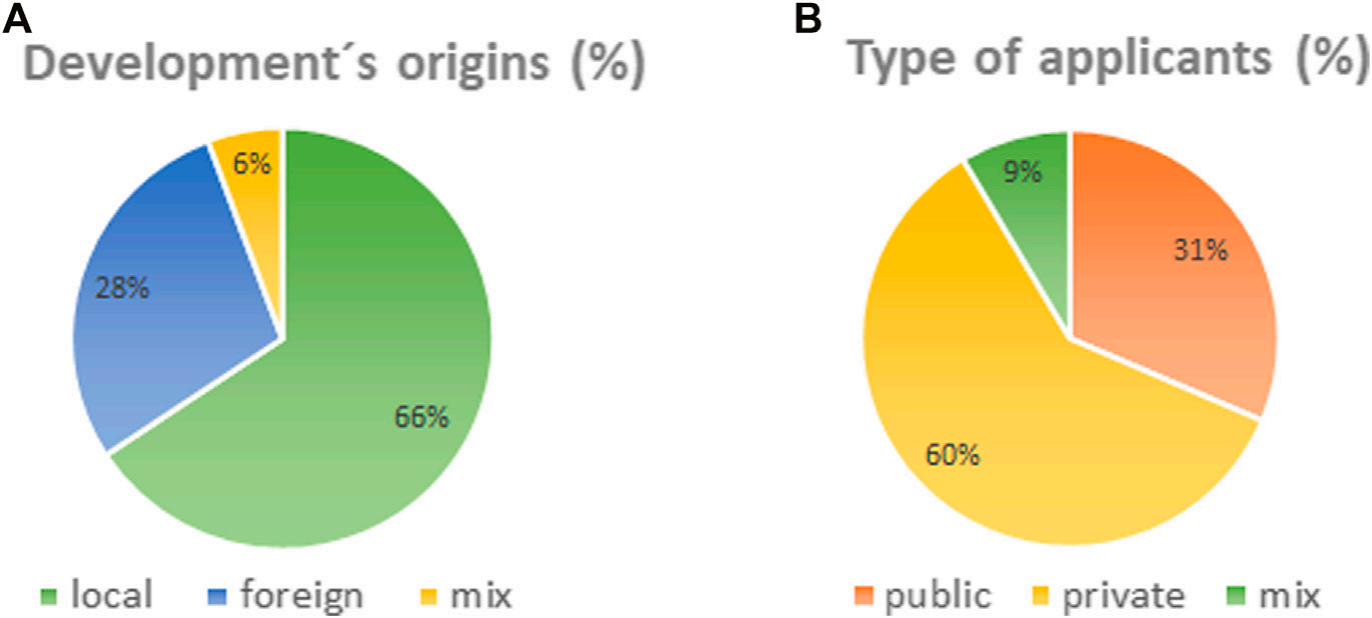
**(A)** Origin of PCI submitted to the Coordination of Innovation and Biotechnology of the National Directorate of Bioeconomy. **(B)** Type of applicants that submitted PCIs.

Additionally, among the cases analyzed, the following results were obtained: between 2015 and 2021 there were 35 PCI cases (Figure 1). The proportion of techniques used was 86% gene editing and 14% others NBT. The queries were made by 66% local developers; 28% foreign developers. Of that percentage, most of the foreign developments presented come from North America and the minority from Europe. Finally, 6% foreign developments were submitted by local companies. Regarding the type of applicants submitting PCIs, 60% were private companies, 31% public institutions and 9% were mixed entities (Figure 2). In contrast to the scenario of Argentina’s development of GMO, the origin of developments and type of applicant, 95% are from foreign origin and 5% from national origin. Regarding NBTs, PCI submitted distribution by type of organism was: 57% crops, 29% animals, and 14% microorganisms. Talking about the state of the developments, 60% of the PCIs were hypothetical products, while the 40% of the PCIs were about real products. As an example of a local product developed in Argentina applying genetic edition, it could be mentioned the reduced enzymatic browning in potato tubers, obtained by a public research institution (González et al., 2020).

### Innovation

In addition to the improvement of the regulations, which came into force in January 2021, and taking into account the analysis of Lewi and Vicién, (2020), other actions were launched to promote the approach of local developers to the knowledge of the regulations and encourage the presentation of their cases through PCIs. A form.^4^ was generated on the website of the Coordination of Innovation and Biotechnology of the National Directorate of Bioeconomy called “Should my product be regulated?” which contains a short questionnaire that developers must complete to make inquiries on issues related to the regulation of GMOs or NBTs.^5^

Another action carried out by the Coordination of Innovation and Biotechnology of the National Directorate of Bioeconomy is the participation of local developers and representatives of the different public/private research institutions to attend virtual meetings (since they were held during the pandemic) with the aim of establishing a direct channel of communication with local developers in order to address relevant issues regarding problems related to regulation and funding that the institutions are currently facing.

Paying attention to the demands of local “Biodevelopers” to have a specific treatment from public policies, the “Argentine Biodevelopment Initiative” has been launched to accompany and strengthen capacities. This space seeks to promote innovation and accompany researchers and developers in the country in the management of activities related to biotechnology by promoting advances in regulatory processes. It seeks to facilitate and organize access to information and the generation of regulatory data to be presented to regulatory agencies.

### International Cooperation

Argentina was the first country to develop specific regulations for the differential treatment of products derived from new breeding techniques. After the official publication of the first resolution in 2015, other countries such as Chile.^6^, Brazil.^7^, Paraguay.^8^ developed their regulations contemplating similar criteria. Subsequently, Colombia.^9^, Guatemala and Honduras.^10^ adopted similar regulatory frameworks, as well as Japan and Israel, which have their regulations in force.

Products derived from NBTs could be considered GMO or not, so there must be a prior analysis. The edge for being considered GM or no GM is the CPB definition of GMO or LMO. When a product derived from NBTs is not under the scope of the CPB GMO definition, in Argentina it is considered a conventional product. Taking the before mention into account, in those cases the Cartagena Protocol on Biosafety should not need to apply to genome editing as these are mutagenic techniques that don’t require CPB oversight, as there is no “new genetic combination”.

Currently, there are several international forums where Argentina actively participates along with other countries. One of the most important events was the participation in the formulation of two international declarations: in 2018, the International Declaration in favor of agricultural applications of precision biotechnology and in 2019 the South Agricultural Council (CAS) Declaration at the WTO (World Trade Organization) in favor of genomic editing techniques. Nevertheless, efforts are still needed in international dialogues, international capacity building and accurate promotion in order to properly adopt these technologies.

## Discussion

This regulation was a pioneer in analyzing products derived from biotechnology using NBTs. The approach based on the analysis of the product obtained (real cases) or to be obtained (hypothetical cases), instead of the technologies used (from the long and dynamic list of technologies called NBTs), allows the regulation to be kept up-to-date and can be applied regardless of the scientific advances that are presented.

The fact of having separated the analysis of NBTs from the rest of the GMO regulations is also a value generated in this update of the regulations. Developers find greater opportunities to approach the regulatory system and make their inquiries. This gives greater predictability to projects, especially those of local development, which always has many difficulties to complete the path of innovation with their products derived from the application of modern biotechnology.

The spirit of the regulation is to contemplate all organisms under the same resolution independently of commercial regulations for GMOs. Also, this regulation gives certainty to the local researchers and developers, and this is observed in the amount of developments and consultations carried out.

## Data Availability

The raw data supporting the conclusions of this article will be made available by the authors, without undue reservation.

## Footnotes

1
https://argenbio.org/recursos/66-estadisticas-isaaa/128-evolucion-superficie-gm.

2
https://www.boletinoficial.gob.ar/detalleAviso/primera/240529/20210208.

3
https://www.cbd.int/doc/legal/cartagena-protocol-en.pdf.

4
https://www.magyp.gob.ar/conabia/.

5
https://www.magyp.gob.ar/conabia.

6
http://www.sag.cl/ambitos-de-accion/aplicabilidad-de-resolucion-ndeg-15232001-en-material-de-propagacion-desarrollado-por-nuevas-tecnicas-de-fitomejoramiento.

7
http://ctnbio.mctic.gov.br/resolucoes-normativas/-/asset_publisher/OgW431Rs9dQ6/content/resolucao-normativa-n%C2%BA-16-de-15-de-janeiro-de-2018?redirect=http%3A%2F%2Fctnbio.mctic.gov.br%2Fresolucoes-normativas%3Fp_p_id%3D101_INSTANCE_OgW431Rs9dQ6%26p_p_lifecycle%3D0%26p_p_state%3Dnormal%26p_p_mode%3Dview%26p_p_col_id%3Dcolumn-2%26p_p_col_count%3D3%26_101_INSTANCE_OgW431Rs9dQ6_advancedSearch%3Dfalse%26_101_INSTANCE_OgW431Rs9dQ6_keywords%3D%26_101_INSTANCE_OgW431Rs9dQ6_delta%3D15%26p_r_p_564233524_resetCur%3Dfalse%26_101_INSTANCE_OgW431Rs9dQ6_cur%3D2%26_101_INSTANCE_OgW431Rs9dQ6_andOperator%3Dtrue.

8
https://conbio.mag.gov.py/media/ckfinder/files/Resolucion%20565%20de%202019.pdf.

9
https://www.ica.gov.co/getattachment/2d02cc52-d1c5-4123-8a5a-aea9ad2ce926/2018R29299.aspx.

10
http://www.senasa.gob.hn/images/ACD/2019/ACUERDO-CD-SENASA-008-2019%20GACETA%2035047.PDF.
